# Effectiveness of Hydrogalvanic Bath on Improving Pain, Disability, and Quality of Life in Individuals with Chronic Nonspecific Neck Pain: A Randomized Controlled Trial

**DOI:** 10.1155/2020/7974816

**Published:** 2020-10-06

**Authors:** Mastour Saeed Alshahrani, Jaya Shanker Tedla, Ravi Shankar Reddy, Faisal Asiri

**Affiliations:** Department of Medical Rehabilitation Sciences, College of Applied Medical Sciences, King Khalid University, Abha, Saudi Arabia

## Abstract

**Background:**

Neck pain is one of the world's leading factors in years lived with disability. Ambiguity in the effect of electrotherapy modalities for the treatment of chronic nonspecific neck pains (CNSNP) needs to be examined further. This study sought to elucidate the effectiveness of hydrogalvanic bath on improving pain, disability, and quality of life among individuals with CNSNP.

**Methods:**

Thirty-four individuals with a diagnosis of CNSNP were selected through convenient sampling and randomly divided into two groups by block randomization. The control group treatment underwent low Transcutaneous Electrical Nerve Stimulation (TENS) and exercise, and the experimental group was subjected to hydrogalvanic bath therapy (HGBT) and exercise. Individuals were evaluated for pain using a visual analog scale (VAS), disability with the Neck Disability Index (NDI), and quality of life with Short Form-36 (SF-36). These measures were applied at baseline and after 12 weeks of treatment.

**Results:**

The pretreatment and posttreatment results for VAS, NDI, and SF-36 were compared for both control and experimental groups. We found that all the three variables showed significant differences between the two time points with *p* < 0.05 in both the groups but the experimental group improvements were more significant than the control group with *p* < 0.05.

**Conclusion:**

Twelve weeks of low TENS or HGBT along with exercises can decrease pain and neck disability and increase the quality of life in individuals with CNSNP. However, HGBT along with exercise has superior effects relative to low TENS along with exercise. This randomized controlled trial was registered in the International Standard Randomized Controlled Trials Number-ISRCTN29695190 and registered on 05/02/2020. This study is a retrospective registration.

## 1. Background

People living in the current era are dependent on viewing screens of electronic gadgets for many needs. Along with this dependence, a lack of exercise and bad posture increases the rate of musculoskeletal problems like neck pain. This was further proven in the 2017 Global Burden of Disease study, where neck pain was recorded among the worldwide leading causes for years lived with disability [1, 2]. Worldwide, the prevalence of neck pain was reported from 15% to 50% by various studies [3, 4]. Neck pain can be attributed to many etiologies like pathological ailments or imbalance in the musculoskeletal system or can be associated with some anatomical structures [5]. However, the majority of neck pain is nonspecific in nature without a connection to any structure [6, 7].

As per the symptom duration, neck pain can be acute (less than six weeks of onset), subacute (between six weeks and three months of onset), or chronic (greater than three months of onset). The prognosis for acute and subacute neck pain is better than that for chronic neck pain [6]. At least 50% of nonspecific neck pain converts to chronic in nature, which is characterized by continuous pain or recurrence of pain [8]. Neck pain is a significant burden on an individual at a personal level: it affects their mood, socialization capacity, work capacity, coping ability, and quality of life [9]. Economically, the person loses their income due to work absence, frequent hospital visits, and the use of medications and treatments to control pain [10]. In the Kingdom of Saudi Arabia, neck pain was reported as one of the most common health issues in many professionals and workers [11–15].

There are a variety of conservative treatment options available for chronic nonspecific neck pain, but many treatment systematic reviews and meta-analyses have provided ambiguous evidence [16–21]. Pharmacological therapy is not very effective in treating chronic nonspecific neck pain; even though pain reduces temporarily with medical management, it leads to many long-term adverse effects and complications [8, 22]. Alternative complementary medical management options like hydrogalvanic bath can be tried to alter this chronic nonspecific neck pain. Galvanic current is traditionally used in physical therapy for reducing pain, enhancing tissue healing, and stimulating denervated muscles [23]. The application of direct galvanic current or galvanic current together with some medications was tried in the context of neck pain, but the results were ambiguous [16, 24].

Traditional galvanic current (direct current) along with sinusoidal current has been rectified to produce diadynamic current (DDC) with a frequency of 50 to 100 Hz. There are five different varieties of DDC: monophase, diaphase, short-period, long-period, and syncopated rhythm [25]. In the recent literature, DDC has been proven to be effective in discopathy, [26] chronic low back pain, [27] heel pain, [28], and myofascial trigger points [29, 30]. Hydrogalvanic bath therapy (HGBT) is a combination of galvanic current/DDC and warm water immersion. The combination of hydrotherapy with medical galvanism makes this hydrogalvanic bath a unique therapeutic option [31]. HGBT has been used previously for the treatment of fibromyalgia [32], ankylosing spondylitis, [33] diabetic angiopathy, rheumatoid and gouty arthritis, [34], and lumbosacral radiculopathy [31]. As per our clinical observation, we found that HGBT was very effective in treating individuals with CNSNP. Unfortunately, the published literature is currently scarce regarding the effect of HGBT in individuals with CNSNP. Thus, the current study aimed to assess the effectiveness of HGBT on pain, disability, and quality of life in individuals with CNSNP.

## 2. Methods

This study adheres to CONSORT guidelines and it was a balanced block, randomized, single blinded, and parallel group study conducted in the Kingdom of Saudi Arabia. This randomized controlled trial was registered in the International Standard Randomized Controlled Trials Number ISRCTN29695190. We used ClinCalc.com for calculating sample size. For participant's enrollment, allocating them randomly to various groups and assigning the participants to the intervention is done by different authors. We recruited 34 individuals (16 males and 18 females) with a diagnosis of CNSNP from outpatient physical therapy clinics of King Khalid University. The individuals were referred to our clinic by orthopedicians/neurologists/general physicians. The study is of a one-year duration and is conducted between 2019 and 2020 years. Eligible patients for inclusion were between the ages of 18 and 70 years with neck pain in the cervical region for more than three months, without any specific structure associated with their neck pain, and with pain radiating to the nuchal line, shoulder, occiput, upper limbs, or neck muscles. CNSNP individuals with any malignancy, neurological issues, headaches with a specific diagnosis, trauma, infections, skin cuts on upper limbs, or any “red flags” [35] of cervical pain were excluded from the study. This study was approved by the scientific research ethics committee of King Khalid University (HA-06-B-001). The included individuals with CNSNP were educated about the study details and provided written informed consent.

### 2.1. Procedure

**Figure 1:**
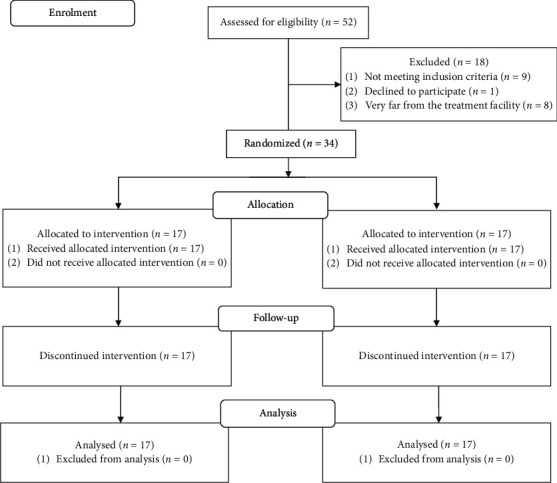
CONSORT flow diagram showing enrollment, allocation, follow-up, and analysis of the participants in the study.

Thirty-four individuals with CNSNP were selected by convenient sampling and randomly divided into two groups, a control group and an experimental group, by block randomization. A random allocation sequence was done by using computer generated random number table. Details of enrollment, allocation, follow-up, and analysis were provided in Figure 1. The control group underwent conventional physical therapy with low TENS and exercise, and the experimental group participated in HGBT and exercise. Each group included 17 individuals. The duration of treatment for both groups was 20 minutes per day, four times a week over a period of 12 weeks for a total of 48 sessions. Along with the 20 minutes of electrotherapy (TENS or HGBT), there is a 15-minute home-based neck exercises, which makes the total duration of treatment 35 minutes. Individuals' demographic data, neck pain onset, location of pain, and area of radiation were noted in the data entry sheet. One of the commonest outcome measures used for assessing neck pain was the Visual Analog Scale (VAS), which is the patient reported measure of pain intensity on a 10-centimeter horizontal line. The Neck Disability Index (NDI) was a specially designed measure of functional disability and it is the most strongly validated and widely used in individuals with neck pain. Short Form-36 (SF-36) measures wellbeing and overall health of the patients along with functional ability and it is a gold standard measurement for assessing quality of life. Individuals were evaluated by a blinded author for pain with VAS, disability with the NDI, and quality of life with SF-36. These outcome measures were applied at baseline and after 12 weeks of treatment.

**Figure 2:**
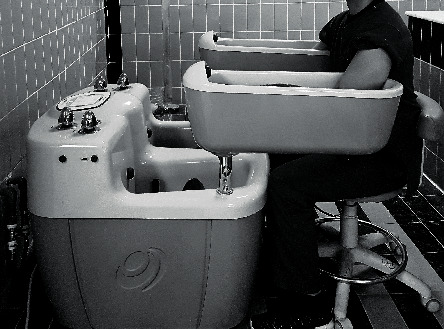
CNSNP patient receiving hydrogalvanic bath therapy.

The HGBT (Electra, Slovak Republic) is a cutting-edge electrotherapy machine containing four tubs (two leg and two hand tubs) with integral electrodes. Stainless steel electrodes measuring 40 × 20 cm in each tank can be connected to the anode or the cathode of the source of the galvanic current [31]. The galvanic bathtubs measure 185 × 80 × 60 cm and are made up of a glass fiber material reinforced with plastic [34]. The individuals upper limbs were freed from clothing and all the metals and any materials like watches, threads, and bands were removed. The individuals were seated on the movable chair near to the bathtub arranged in such a way they immersed both hands in the tanks underwater (Figure 2). We used a *diaphase fixed* variety of DDC and the intensity was slowly raised to the level of maximum patient comfort with a resulting strong vibration sensation in the body part immersed.

The conventional group received low TENS (Sonopuls 692, Enruf Nonius) with a frequency between 5 and 10 Hz. Subjects in both groups performed isometric neck exercises and postural correction strategies as a part of home-based neck exercise program. These exercises were initially demonstrated by professionals at the hospital and once they are confident enough, then they were asked to perform these exercises at home. A video recording of their own exercises was given as further reference to them for performing exercises alone at home. The subjects sat in a chair to perform isometric neck exercises, keeping their shoulders relaxed and heads straight. The subjects pressed the palms of their hands against their foreheads, resisted with their neck muscles, held for 10 seconds, and subsequently relaxed. They performed these exercises in flexion, extension, and side bending, left and right. The subjects performed cervical retraction and scapular retraction exercises as a part of postural correction strategies. For cervical retraction exercises, subjects sat comfortably in a chair maintaining a comfortable neutral position; the subject were asked to pull the head and neck into a position in which the head is aligned more directly over the thorax (chin) while the head and eyes remain level for 10 seconds. For scapular retraction exercises, the subjects were asked to take a deep breath and expand the chest. Then the patient was asked to move his or her shoulders backward bringing the scapulae together for 10 seconds. All the exercises were repeated 10 times in a session and two sessions per day were performed.

### 2.2. Data Analysis

We analyzed data using the Statistical Package for the Social Sciences version 20 software program (SPSS 20.0; IBM-SPSS Inc., Armonk, NY, USA). The level of significance was set at 0.05 with a confidence interval of 95%. The normal distribution of the variables was assessed using the Shapiro–Wilk test. Demographic and outcome variables for univariate analyses were completed by using descriptive statistics. Since the variables are skewed in distribution, we used the Wilcoxon signed-rank test to elucidate the differences of the dependent variables within the groups over time. The Mann–Whitney U test determined the demographic and clinical variable differences between groups.

## 3. Results

In this study, 34 individuals with CNSNP were equally divided into control and experimental groups. Each group's demographic characteristics like age, gender, height, weight, and body mass index are presented in Table 1 (mean ± standard deviation). We measured pain with VAS, disability with NDI, and quality of life with SF-36. All these measurements were done at baseline and the 12th week.

At baseline, anthropometric values and outcome measures were compared between the control and experimental groups, and we found that there were no significant differences between the groups with *p* > 0.05 for all the tested variables. The details of these compared parameters with their mean ± standard deviation and *p*-values are presented in Table 1.

The changes from pretreatment to posttreatment for VAS, NDI, and SF-36 were compared for both the control and experimental groups. We found that all three variables showed significant differences between pretreatment and posttreatment with *p* < 0.05. The details of the pre- versus posttreatment (mean ± standard deviation) and their *p*-values are presented in Table 2.

Similarly, the mean differences in improvements according to VAS, NDI, and SF-36 were compared for both control and experimental groups. We found that all the three variables showed significant changes for mean change between pretreatment and posttreatment between the control and experimental groups with *p* < 0.05. The details of these differences including mean ± standard deviation and *p*-values are presented in Table 3. There were no adverse effects or harms reported by any of the interventions used in this study.

## 4. Discussion

The current study sought to elucidate the effect of HGBT on individuals with CNSNP. To our knowledge, this is a first-of-its-kind randomized controlled trial conducted among individuals with CNSNP using a hydrogalvanic bath. Both the control and experimental groups' treatments were shown to be effective in decreasing pain, disability, and quality of life, but the experimental treatment findings suggested greater effectiveness relative to the conventional treatments.

Borodulina et al. previously published a similar study on 60 chronic lumbosacral radiculopathy patients. Three months of intervention led to statistically significant changes in VAS (<0.01), Douleur Neuropathique 4 questionnaire (<0.01), pain detection (<0.01), Oswestry Disability Index (<0.01), and Beck Depression Inventory (<0.01) [31]. In our study, three months of HGBT also promoted similar improvements in VAS, NDI, and SF-36 with a significance value of less than 0.001.

Elsewhere, Mucha conducted a study among 60 individuals with chronic epicondylitis for six weeks. The control group received interferential therapy and the experimental group received hydrogalvanic and temperature-increasing bath therapy. The study measured pain, elbow active joint range of motion, and grip strength. After the intervention, the experimental group showed significant improvements in all these outcome measures with *p* < 0.01 [36]. In our study as well, we observed similar improvements as compared to with the conventional low TENS treatment for all the outcome measures with *p* < 0.001.

DDC is produced by the electronic processing of sinusoidal, symmetrical, and alternating currents. By half-wave and full-wave rectification, *monophase fixe* and *diaphase fixe* currents are produced, respectively [37]. The major therapeutic effects of these low-frequency DDCs include pain relief, decreased inflammation and swelling, increased blood circulation by vasodilatation, and the facilitation of tissue healing [25]. These effects can be linked to improvements in the experimental group. The primary explanation for pain relief produced by DDC can be explained by the pain gate theory and by physiological responses produced in the tissues by way of stimulation of sensory and motor nerves. Another hypothesis for the decrease in pain provoked by DDC stimulation is an increase in the volume of polypeptides and endorphins accountable for pain relief [26, 38, 39]. Some of the recent literature has stated that there is a sympathetic system abnormality in individuals with CNSNP [40, 41]. The DDC of diaphase fixe variety used in HGBT is hypothesized to decrease the increased sympathetic tone in individuals with CNSNP [25].

The improvements in the experimental group can be further attributed to the effects of HGBT, which include analgesic, anti-inflammatory, decongestant, improved microcirculation, and reduced sensory impairment activities [34, 42]. Warm water in the bath can further enhance the effect of DDC and the buoyancy effect of the water decreases the weight of the upper extremities on the axial skeleton, which further decreases stress on the muscles.

The lack of large sample size, the dearth of treatment follow-up, and the shortage of multicentric application are some of the major drawbacks in our study. Future research with larger sample sizes and long-term follow-up in a multicentric basis is warranted.

## 5. Conclusion

Twelve weeks of low TENS or HGBT along with exercise can decrease pain and neck disability and increase the quality of life in individuals with CNSNP. However, HGBT along with exercise had superior effects when compared with low TENS along with exercise.

## Figures and Tables

**Table 1:** Demographic and baseline characteristics of the study population.

Variable	Experimental mean ± SD	Control mean ± SD	*p* value
Age (yrs.)	47.65 ± 7.71	49.47 ± 6.22	0.496
Gender (M : F)	07 : 10	09 : 08	0.345
Height (mt)	1.67 ± 0.08	1.63 ± 0.12	0.205
Weight (kg)	73.35 ± 7.50	72.12 ± 8.96	0.683
BMI (kg/mt^2^)	26.57 ± 4.14	27.45 ± 5.24	0.634
SF-36	37.71 ± 3.80	40.47 ± 5.72	0.106
NDI (neck disability)	28.29 ± 2.71	30.59 ± 7.00	0.708
VAS (pain intensity)	6.53 ± 1.23	6.41 ± 1.37	0.658
Pain radiation			
Neck pain	13	14	0.234
Arm pain	04	03	0.524

BMI: body mass index; NDI: Neck Disability Index; VAS: visual analog sale.

**Table 2:** Comparison of outcome measures differences pretreatment versus posttreatment for both experimental and control groups.

Group	Outcome measures	Pre		Post		*p* value
		Mean	SD	Mean	SD	
Experimental	SF-36	37.71	3.80	47.47	5.48	<0.001
	NDI	28.29	2.71	19.18	4.33	<0.001
	VAS	6.53	1.23	2.24	1.48	<0.001

Control	SF-36	40.47	5.72	40.77	5.70	0.025
	NDI	30.59	7.00	29.12	6.90	<0.001
	VAS	6.41	1.37	4.77	1.25	<0.001

**Table 3:** Comparison of outcome measures mean differences both experimental and control groups.

Outcome measures	Experimental		Control		*p* value
	Mean	SD	Mean	SD	
Pre- versus post- differences in SF36	9.765	4.724	0.30	1.8109	<0.001
Pre- versus post- differences in NDI	9.118	3.5157	1.47	1.7321	<0.001
Pre- versus post- differences in VAS	4.294	1.2127	1.647	0.8618	<0.001

SD: standard deviation, SF-36: Short Form -36, NDI: Neck Disability Index, and VAS: visual analog scale.

## Data Availability

All data are available at the Department of Medical Rehabilitation Sciences (College of Applied Medical Sciences) upon request to the first author Mastour Saeed Alshahrani (msdalshahrani@kku.edu.sa).

## Abbreviations

CNSNP: Chronic nonspecific neck pains
TENS: Transcutaneous electrical nerve stimulation
HGBT: Hydrogalvanic bath therapy
VAS: Visual analog scale
NDI: Neck disability index
SF-36: Short form-36
DDC: Dia dynamic current.
